# Efficacy of Anti-Inflammatory Therapy in a Model of Acute Seizures and in a Population of Pediatric Drug Resistant Epileptics

**DOI:** 10.1371/journal.pone.0018200

**Published:** 2011-03-28

**Authors:** Nicola Marchi, Tiziana Granata, Elena Freri, Emilio Ciusani, Francesca Ragona, Vikram Puvenna, Quingshan Teng, Andreas Alexopolous, Damir Janigro

**Affiliations:** 1 Department of Neurological Surgery, Cleveland Clinic Foundation, Cleveland, Ohio, United States of America; 2 Department of Molecular Medicine, Cleveland Clinic Foundation, Cleveland, Ohio, United States of America; 3 Department of Cell Biology, Cleveland Clinic Foundation, Cleveland, Ohio, United States of America; 4 Cerebrovascular Research, Cleveland Clinic Foundation, Cleveland, Ohio, United States of America; 5 Epilepsy Center, Cleveland Clinic Foundation, Cleveland, Ohio, United States of America; 6 Istituto Neurologico Besta, Milan, Italy; Biological Research Center of the Hungarian Academy of Sciences, Hungary

## Abstract

Targeting pro-inflammatory events to reduce seizures is gaining momentum. Experimentally, antagonism of inflammatory processes and of blood-brain barrier (BBB) damage has been demonstrated to be beneficial in reducing status epilepticus (SE). Clinically, a role of inflammation in the pathophysiology of drug resistant epilepsies is suspected. However, the use anti-inflammatory drug such as glucocorticosteroids (GCs) is limited to selected pediatric epileptic syndromes and spasms. Lack of animal data may be one of the reasons for the limited use of GCs in epilepsy. We evaluated the effect of the CG dexamethasone in reducing the onset and the severity of pilocarpine SE in rats. We assessed BBB integrity by measuring serum S100β and Evans Blue brain extravasation. Electrophysiological monitoring and hematologic measurements (WBCs and IL-1β) were performed. We reviewed the effect of add on dexamethasone treatment on a population of pediatric patients affected by drug resistant epilepsy. We excluded subjects affected by West, Landau-Kleffner or Lennox-Gastaut syndromes and Rasmussen encephalitis, known to respond to GCs or adrenocorticotropic hormone (ACTH). The effect of two additional GCs, methylprednisolone and hydrocortisone, was also reviewed in this population. When dexamethasone treatment preceded exposure to the convulsive agent pilocarpine, the number of rats developing status epilepticus (SE) was reduced. When SE developed, the time-to-onset was significantly delayed compared to pilocarpine alone and mortality associated with pilocarpine-SE was abolished. Dexamethasone significantly protected the BBB from damage. The clinical study included pediatric drug resistant epileptic subjects receiving add on GC treatments. Decreased seizure frequency (≥50%) or interruption of *status epilepticus* was observed in the majority of the subjects, regardless of the underlying pathology. Our experimental results point to a seizure-reducing effect of dexamethasone. The mechanism encompasses improvement of BBB integrity. Our results also suggest that add on GCs could be of efficacy in controlling pediatric drug resistant seizures.

## Introduction

Drug-resistant seizures pose a formidable challenge for drug development. Recently, the Consensus Proposal by the *ad hoc* Task Force of the International League against Epilepsy Commission on Therapeutic Strategies pointed out that drug resistance is “*a dynamic process rather than a fixed state*” and in spite of the available therapeutic interventions for seizure disorders, the incidence of epilepsies and mortality associated to status epilepticus remain elevated. Moreover, treatment of childhood epilepsy is of particular interest since it is widely accepted that “seizures beget seizures”; thus, patients who experience seizures early in life are more likely to develop an epileptic pathology later in life. In spite of the available therapeutic interventions for seizure disorders, the incidence of epilepsies and mortality associated to status epilepticus remain elevated [1].

Clinical [2]–[4] and translational [4]–[7] studies have shown that the gatekeeper at the brain-systemic circulation interface, the blood-brain barrier (BBB), may be a crucial player in epilepsy [8]. A role of inflammation and BBB dysfunction in the pathophysiology of human epilepsies is suspected. Histological studies have shown BBB dysfunction in human epileptic tissue [9]–[11]. In addition, DWI or post-contrast FLAIR MRI changes corresponded to the location of EEG activity in patients with partial status epilepticus or focal epilepsy [12]–[18]. Increased CSF albumin, an index of increased BBB permeability, was also reported at the time of EEG slow-wave activity [19].

From a clinical standpoint, a role of inflammation and BBB dysfunction in the pathophysiology of drug resistant epilepsies is suspected. However, the use of anti-inflammatory molecules such as glucocorticosteroids (GCs) is for the most part limited to infantile spasms, Landau-Kleffner or Lennox-Gastaut syndromes and Rasmussen's encephalitis. Their efficacy in drug resistant forms of pediatric epilepsies is still anecdotal [20]–[22]. A previous report [23] demonstrated the utility of GCs in other epilepsy syndromes, but no specific mechanism of action was proposed.

From an experimental standpoint, there is evidence that inflammation, having significant effects on BBB integrity, affects normal brain function and contributes to the pathophysiology of seizures [4],[6],[11],[24],[25]. These concepts have been expanded to the pilocarpine model of seizure [24], [26]. Pilocarpine induces acute peripheral pro-inflammatory changes leading to BBB leakage prior to SE. These studies also demonstrated that pilocarpine is relatively impermeant across the BBB and it is therefore unlikely that this cholinergic drug act directly, or only, on neurons [24], [26]. The alternative model based on pilocarpine and lithium given in combination also encompasses pro-inflammatory events. Of interest is the fact that the effects of lithium, which has a powerful immunological action [27], were independent on the schedule of administration. Lithium potentiated pilocarpine's action even when given after pilocarpine, which sheds uncertainty on the concept of lithium priming the brain cholinergic system. Previous studies have shown that pharmacological manipulation of intravascular inflammation and restoration of BBB integrity reduces seizure onset in the pilocarpine model of TLE [28], [29].

These data suggest that the inflammatory process and loss of BBB integrity may play a pathological role in a broad spectrum of epilepsies. We further evaluated the effect of anti-inflammatory agents in experimental seizures, and tested the efficacy of gluco-corticosteroids. We evaluated the efficacy of anti-inflammatory treatments in drug resistant pediatric epilepsies, excluding those conditions already known to benefit from steroidal treatment, *i.e.* West, Landau-Kleffner, Lennox-Gastaut syndromes and Rasmussen's encephalitis [20]–[22], [30]. We analyzed the response to gluco-corticosteroids, or ACTH, in a pediatric population and analyzed the results to develop a hypothesis that also takes into account data obtained from animal experiments where rats were exposed to convulsive doses of the cholinergic agonist pilocarpine.

The justification for extrapolating data obtained from pilocarpine-induced SE to drug resistant epilepsy may be considered inappropriate and one should ideally compare human data to pilocarpine-treated chronic rats who do not respond to AED. Thus, two points of asymmetry can be found in the current study, one related to chronicity of seizures in humans vs. acute nature of BBB disruption-induced seizures, as well as the issue of human epileptic vs. normal brain induced to seize. In fact, to segregate and study drug resistant rats would constitute the best animal correlate of human multiple drug resistance to antiepileptic drugs. However, recent experimental findings suggested that correlates of acute seizures (*e.g.*, as triggered by iatrogenic BBB disruption or pilocarpine) are not dissimilar from chronic seizures. For instance, seizures acutely induced by intrarterial mannitol have EEG features similar to pilocarpine seizure and the histological and immunohistochemical tracts of acute seizures (*e.g.*, cerebrovascular damage) are similar to the ones observed in the chronic epileptic human brain [31].

## Results

### Efficacy of dexamethasone: rat study

We first evaluated whether dexamethasone prevents the onset of pilocarpine-induced seizures in rats. We also quantified seizure-induced mortality. Experimental details are shown in Figure S1. These effects are compared to those previously obtained using an IL-1 receptor antagonist [29].

**Figure 1:**
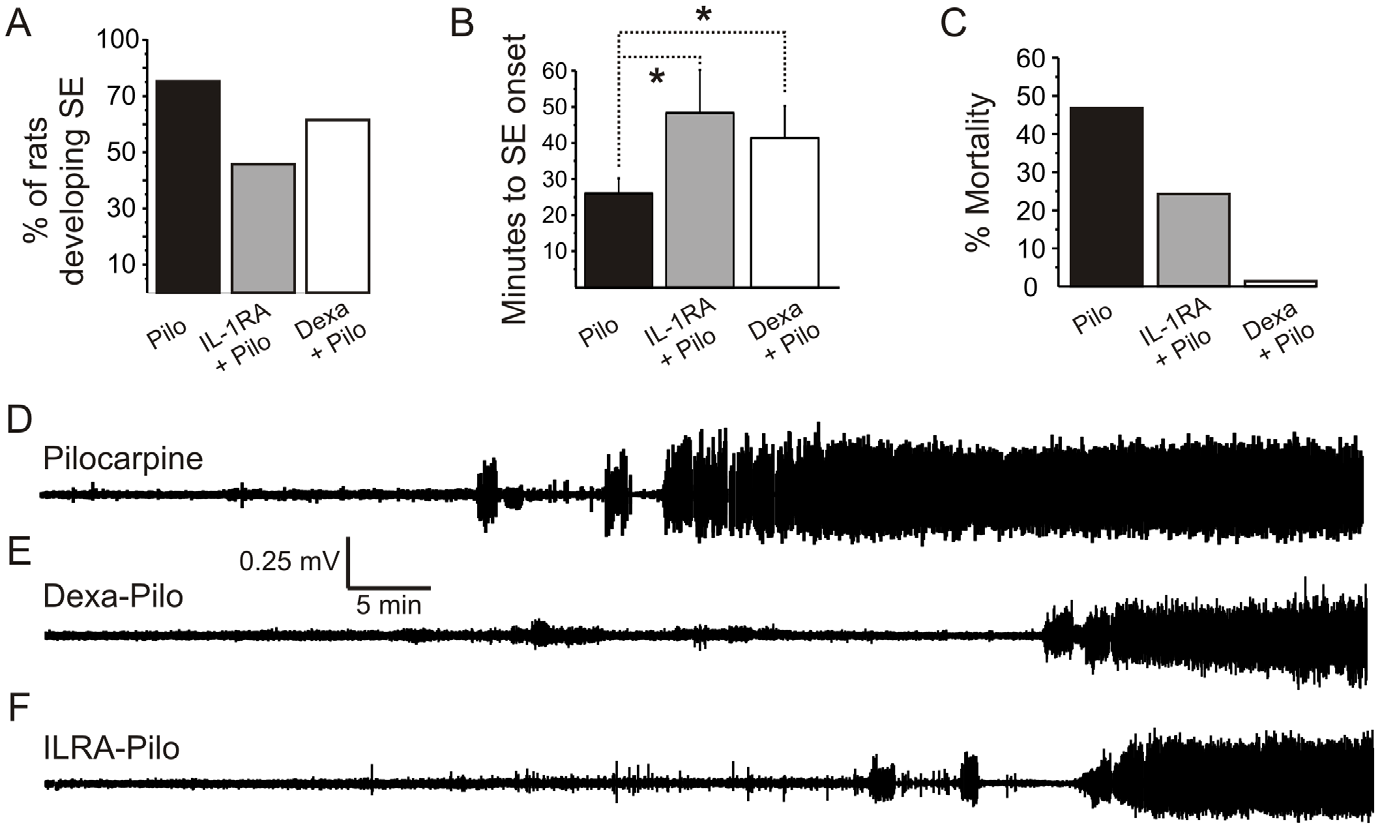
Effects of dexamethasone on pilocarpine-induced SE. (A) The number of rats experiencing SE was reduced by anti-inflammatory treatments. Data are compared to IL-RA treatments [29]. (B) When SE developed, its onset was delayed (control vs. IL1ra p = 0.03; control vs. Dexa p = 0.04). (C) Twelve hours mortality associated with pilocarpine seizures was decreased by IL-RA and abolished by dexamethasone (p = 0.02). To attest the efficacy of treatment on survival, seizures were not stopped using barbiturates. (D-E) Examples of EEG recordings show that SE in treated animals was of lesser intensity compared to pilocarpine alone (see also Figure S2). Data are relative to n = 15 rats / group. Asterisks: p<0.05, by paired t-test and Fisher test.

Figure 1 shows typical EEG traces recorded after injection of pilocarpine alone (D), after dexamethasone pretreatment (E), or after blockade of IL-1β receptors (F). The average results are shown in Figure 1A-C. Note that pilocarpine caused a stereotyped response consisting of an initial burst of action potentials followed by a full-blown and persistent SE. When anti-inflammatory treatment preceded exposure to the convulsive agent (see Figure S1), the number of rats developing develop seizures was reduced (Figure 1A). When SE developed, the time of onset was significantly delayed compared to untreated controls (Figure 3C). Mortality associated with SE was significantly decreased by dexamethasone (p = 0.02, Fisher test).

**Figure 2:**
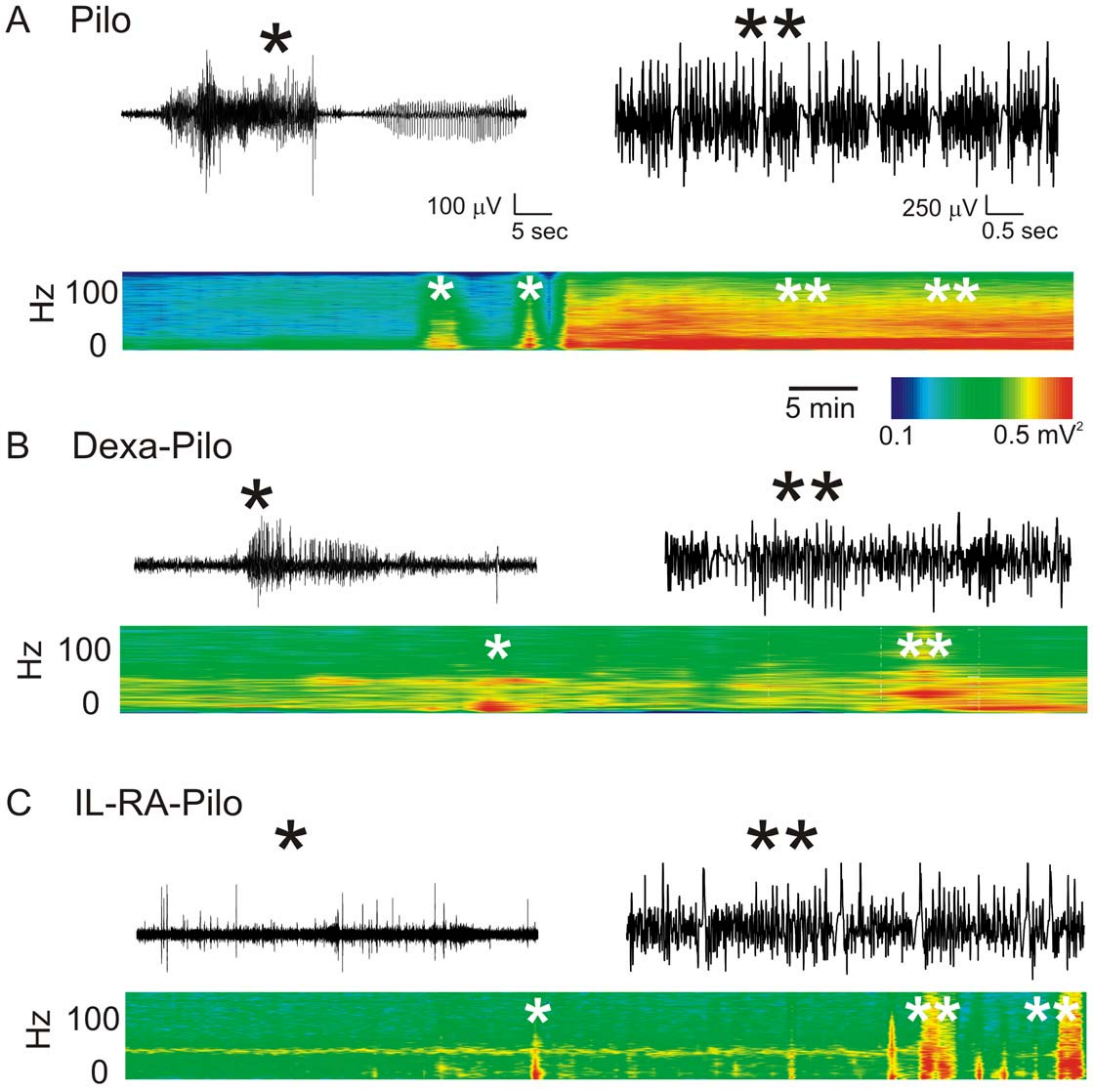
Time-joint frequency analysis of EEG recordings. (A–C) S*ingle asterisk* refers to the first seizure episode. The *double asterisk* shows the maximal electrographic and behavioral seizures observed under any given condition. The actual EEG recordings are also shown. Time-joint frequency plots show a reduction of seizure intensity (frequency and amplitude domains, *color coded*) in treated animals compared to pilocarpine alone. Data shown refer to 2 hours of EEG recordings. See also Figure S2 for peak area distribution and instantaneous frequency analysis.

Time-joint frequency analysis was performed to examine changes not immediately apparent by EEG inspections. Note that the early burst clusters (*single asterisks* in Figure 2) were reduced in amplitude and frequency in animals pre-treated with either dexamethasone or IL1-RA. Severity of SE was also reduced in treated rats (frequency and amplitude distributions) as shown in Figure S2.

### Evaluation of blood-brain barrier damage

**Figure 3:**
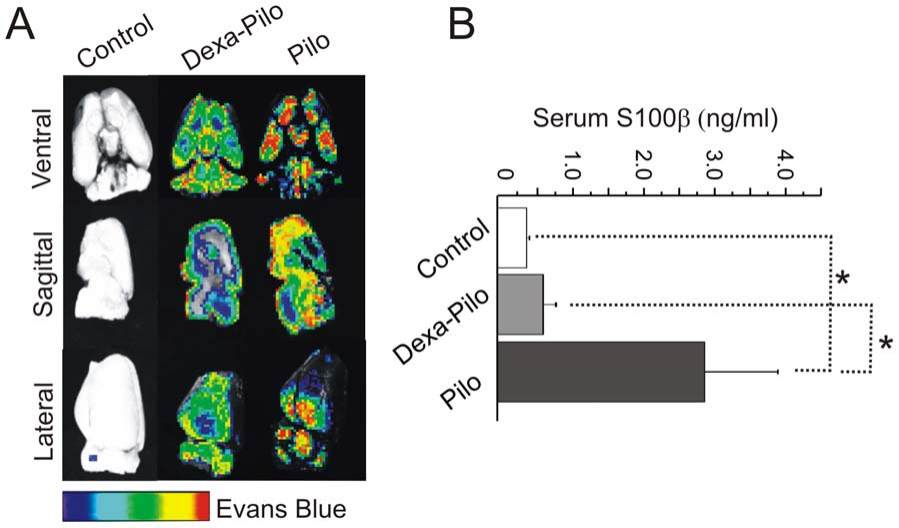
Dexamethasone reduces BBB damage in pilocarpine-treated rats. BBB integrity was assessed by Evans blue (A) and serum S100β (B) measurements. Evans blue is an indicator of paracellular leakage while S100β is a surrogate serum marker of the integrity of the cerebrovascular endothelial interface. Both methods revealed a reduction of pilocarpine-induced BBB damage in dexamethasone-pretreated animals (DEXA-PILO). Similar efficacy was previously reported for IL1-RA (see [29]). The *asterisks* refers to p<0.05 by paired t-test, n = 5 rats per group.

We subsequently compared the integrity of the BBB in animals treated with pilocarpine and in those pre-treated with dexamethasone (Figure 3). We have previously demonstrated that pre-treatment with IL-RA prevents BBB damage and reduces seizure occurrence [29]. BBB integrity was evaluated by visualization of Evans blue extravasation and by measurements of serum levels of S100β [32]. Note the significant increase of serum S100β levels and Evan's Blue signal measured at onset of SE (Figure 3). Both S100β levels and Evan's Blue signal were comparable to control when seizures were prevented or reduced by dexamethasone. These results are in agreement with animal studies previously published [5], [33]. Remarkably, a decrease in MRI FLAIR hyperintensity concurrent with seizure reduction was observed in patients after corticosteroid treatment (see text below and Figure 6).

### Serological correlates of dexamethasone efficacy in rats

**Figure 4:**
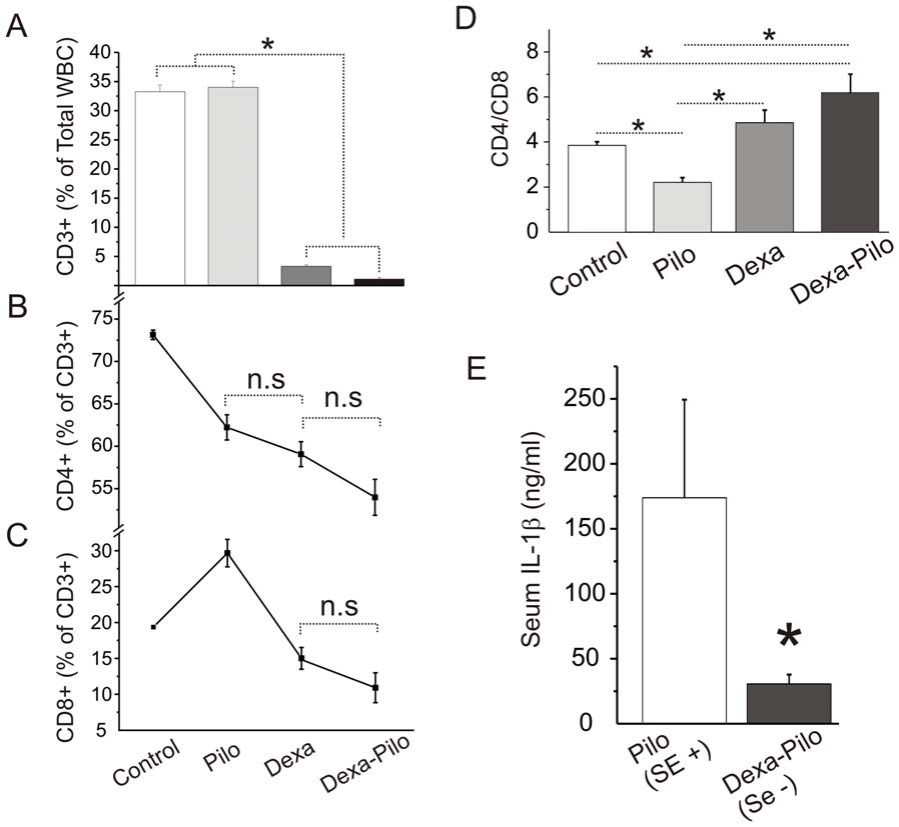
Hematologic and serologic changes after dexamethasone treatment in rats.

We analyzed circulating white blood cells (WBCs) to determine the level of T-lymphocyte activation after pilocarpine or after dexamethasone followed by pilocarpine. The most important effect of dexamethasone was a drastic reduction in the number of circulating T-cells (CD3+, Figure 4A). Among the remaining CD3+ cells, the relative percentage of CD8+ subpopulation was significantly affected by dexamethasone (Figure 4C). However, the latter change may be negligible owing the drastic reduction of total number of circulating T-cells (Figure 4A). A change in CD4:CD8 ratio consistent with an inflammatory activation was seen after pilocarpine treatment (Figure 4D, see also [26], [34]). Dexamethasone significantly reduced the levels of serum IL1-β (Figure 4E).

### Efficacy of gluco-corticosteroids or ACTH: human study

**Figure 5:**
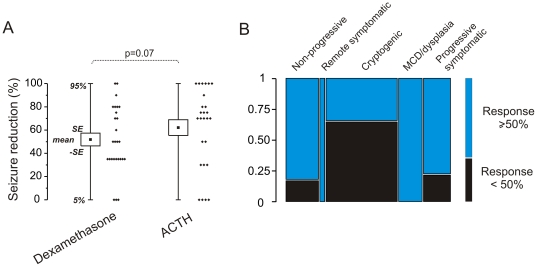
Efficacy of dexamethasone and ACTH in drug resistant pediatric epilepsy. Data relative to the efficacy of methyl-prednisolone and hydrocortisone are presented in Figure S3. (**A**) A total of 53 treatments were evaluated**.** Treatments were administered as described in the Methods and Table S1. Seizures were assessed by behavioral and EEG observations. The values reported refer to decrease in seizure burden compared to baseline EEG seizure quantification. T-test was used to assess significance. (**B**) Mosaic plot showing the correlation between etiology of epilepsy and likelihood of a response ≥50%. Note that etiology did not always predict response. Noteworthy, dysplasia and other non-encephalopathic diseases responded to the treatments. Cryptogenic seizures were least affected. Bar width is proportional to the number of observations. Colors refer to the response as indicated in the *inset*.

We investigated the efficacy of add-on glucocorticosteroids (GCs) or ACTH treatment in patients affected by drug-resistant epileptic seizures (see Table S1). Inclusion criteria are described in the Methods. For the whole study (Figures S3 and S4) we reviewed data from 43 patients (24 females and 19 males, see Methods) and a total of 92 GC treatments. The data presented in Figure 5 refer to 15 patients and 53 treatments with dexamethasone or ACTH. Mean age at GCs or ACTH treatment was 6.4 ± 5.2 years. Etiology and type of epilepsy are summarized in Table S1. First seizure occurred between a few days after birth and 13 years of age (mean ± SE: 2 years ± 8 months). Table S1 shows patients' AED regimens and, in bold, the AED co-administrated with GCs or ACTH.

Figure 5A shows the efficacy of dexamethasone or ACTH treatments. Overall, dexamethasone or ACTH significantly reduced occurrence of seizures. Overall, the response was variable ranging from complete reduction of seizures to no benefit from the treatment. The mosaic plot in Figure 5B shows the distribution of the overall efficacies (set as ≥50%) across different etiologies. The overall results obtained with three different GCs (dexamethasone, methylprednisolone and hydrocortisone) are shown in Figure S3. The analysis of efficacy with regard to the type of epileptic syndrome shows that the best responders were patients with focal seizures (Figure S3C). There was no correlation between seizure history (years) and response to treatment (Figure S3D). The latter result is also shown in the multivariate analysis in Figure S4. We observed common side effects associated with administration of GCs (*e.g.,* increased body weight, anxiety and insomnia). Other side effects were only marginal and did not require cessation of therapy. Only in few cases (5%) GCs were suspended due to changes in coagulation, alteration of blood electrolytes or glycemia.

## Discussion

Our results have shown that dexamethasone reduces the number of rats experiencing status epilepticus (SE) and abolishes mortality. The mechanism by which dexamethasone lessens pilocarpine seizure burden encompasses improved BBB function. This was shown by analysis of dye and marker extravasation in treated vs. untreated animals. We also studied the efficacy of add-on gluco-corticosteroids in a population of pediatric drug resistant patients excluding those syndromes known to be responsive to GCs and ACTH (L-G, L-K, West or Rasmussen's). The effect was beneficial regardless of the pathology and epileptic syndrome. We have also reported a selected case where a decrease in FLAIR signal was associated with seizure reduction. Previous studies have shown that FLAIR hyperintense regions or regions of gadolinium enhancement correspond to sites of BBB leakage [9], [11]–[14], [18], [35].

### Anti-inflammatory therapy: a human-experimental parallel

This manuscript presents findings in a format where clinical data are presented together with animal results. We believe that this is appropriate because: 1) there is a recognized urgency to provide rapid therapeutic advancement by comparing clinical and laboratory results [1], [36]; 2) the anecdotal use of corticosteroids in clinical epilepsy has recently expanded (see below), but its full potential for widespread use is limited by the lack of scientific validation of their use. Our study compared the efficacy of glucocorticosteroids and ATCH: 1) in patients across a wide spectrum of epileptic etiologies and syndromes, 2) with the exclusion of those syndromes known to be steroid responsive (i.e. L-G, L-K, West or Rasmussen's). We have also compared the efficacy of steroids in patients with the effects observed in the pilocarpine model. Previously, we and others demonstrated that direct inhibition of leukocyte-mediated blood-brain barrier disruption, comparable to the effects of GCs, prevents SE in this model [28], [29].

We are aware that there are significant limitations in our human study. A major issue is its *non*-randomized, *no*n-placebo nature. In addition, there was no standardized dosage or regimen for corticosteroid treatment. We believe that these are not insurmountable limitations for a preliminary, mechanism-related study because the patients enrolled acted as their own controls. These patients failed at least three AED medications and were exposed to all traditional anti-epileptic care that benefits most of their drug-respondent counterparts. Thus, while use of a placebo arm is mandatory to evaluate once and for all whether anti-inflammatory therapy is a valid AED alternative, our studies have shown their utility at least as an add-on maneuver. The data provided will hopefully lead to a randomized clinical trial.

Animal data were obtained exclusively from adult rats; this does not appear to be a crucial limitation since patient age ranged from a few months to adolescence and no differences in GCs or ACTH effect were observed with regards to age. Finally, patients received a combination of GCs and anti-epileptic drugs (Table S1) while rodents did not.

### Non-neuronal mechanisms of epilepsy: role of the blood-brain barrier

There are several lines of evidence suggesting that the blood-brain barrier could be a valid adjunctive target for anti-epileptic drug therapy. Animal studies that have shown that breaching the BBB is a reliable mean towards decreased seizure threshold [11] or seizure development [4], [6]. More importantly, these animal studies have been supported by concurrent clinical data showing that blood-brain barrier disruption (BBBD) causes seizures in human subjects [4] and that BBBD has a causative link with post-traumatic epilepsy [3]. We have previously demonstrated that prevention of BBB failure reduces seizure onset [29]. The data presented herein further support a pharmacological approach aimed to prevent BBB failure or restore BBB integrity.

There are important clinical correlations resulting from our findings. First and foremost is the fact that we have shown that the efficacy of corticosteroids in pediatric epilepsy is not limited to epileptic encephalopathies, such as infantile spasms and Rasmussen encephalitis [25], [37], [38]. Rather, the effects seemed to be pronounced in focal epilepsy, including those due to focal dysplastic lesions (Figures 5 and Figure S3). This is consistent with the results of recent studies suggesting the efficacy of corticosteroids in focal and generalized epilepsy [20]–[22] of different etiologies. The impact of corticosteroids on seizures during pre-surgical subdural grid EEG monitoring in drug-resistant children was recently demonstrated. A reduced seizure frequency was found in dexamethasone-treated patient compared with untreated [39].

The results of our experimental study support the hypothesis that inflammatory mechanisms and BBB damage could contribute to seizure generation and severity [40], [41]. We confirmed the pattern of WBC activation in the experimental model used [29]. Interestingly, in rats pre-treated with dexamethasone, we observed a decrease in SE severity (Figures 1 and 2) at time of decreased number of circulating T-cells (Figure 4) and reduction of BBB damage (Figure 3).

**Figure 6:**
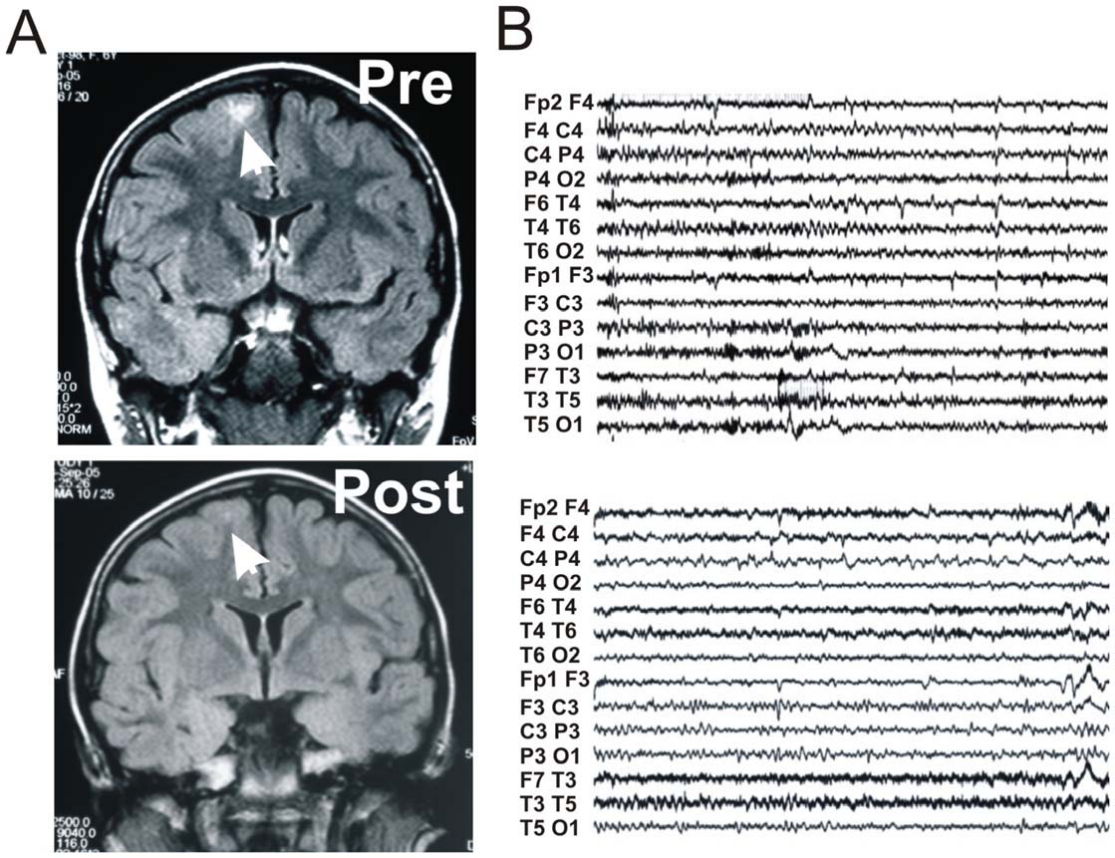
Radiologic indices of successful treatment with dexamethasone. The seizure reducing effect of dexamethasone (B) was paralleled by a decrease in FLAIR hyperintensity.

The efficacy of corticosteroids observed in patients supports the hypothesis that seizures of different etiologies (including those due to congenital malformations or acquired brain damage) may be aggravated by inflammatory mechanisms and BBB disruption. The hypothesis linking BBB damage to seizures is in agreement with histological studies that have shown BBB dysfunction in human epileptic tissue and with MRI studies showing changes corresponding to the location of EEG activity in patients with partial status or focal epilepsy [11], [12], [14]–[18], [25]. Our clinical study design did not include the systematic evaluation of MRI before and after treatments, nevertheless the decrease in FLAIR hyperintensity concurrent with seizure reduction after steroid treatment (Figure 6) suggests the hypothesis that restoring BBB integrity may be one of the mechanisms involved in the antiepileptic action of corticosteroids. A prospective *ad hoc* MR study is mandatory to confirm or disprove this notion.

### Experimental approximation of clinical epilepsy

One obvious issue is that we used only pilocarpine as a model of seizures. This is not, in our opinion, a crucial limiting factor inasmuch that this model has been historically used to model human disease and also because several features of this model have common traits with human epilepsy [42], [43]. For example, it was shown that pilocarpine seizures cause cerebrovascular changes which are consistent with those seen on MRI of patients [43]. These consist of homeostatic failure leading, in patients, to MRI hyperintensity and in animals to increased extravasation of intravascular (Evans blue) or cerebral-to-serum (S100β) indicators (Figure 3). In addition, pilocarpine induces seizures by an unexpected immunologic activation mediated by specific receptors and adhesion molecules [28], [29]. These same molecules are the target of corticosteroids, and depletion of these adhesion molecules appears to protect against pilocarpine or clinical seizures. While the link between steroidal efficacy and BBB needs to be fully demonstrated in patients, we believe that the results we present herein are adequate to warrant an in-depth investigation of the BBB-corticosteroid-epilepsy relationship.

If BBB leakage is the main initiator of seizures, and if BBB repair is protective against neurological disease, which are the mechanisms involved? The experimental evidence provided herein, together with previous findings [25], [26], [28], point to an inflammatory-induced damage of the BBB. Corticosteroids have a profound effect on human and rodent BBB permeability [25], [33], [44]–[47]. Moreover, corticosteroids have an impact on the number of circulating T-cells. Downstream signaling by IL1-β appears to be a common thread in pilocarpine-induced seizures (this paper and [25], [29], [48]). It therefore seems reasonable to assume that the immune system acts in concert to produce BBB leakage and seizures, while the counteraction of corticosteroids has the opposite effect [48]. These results further suggest that seizures and inflammation belong to the same chapter of neuro-immunology, as also shown recently [49].

An important correlate of our findings is the fact that animal data strongly suggest that anti-inflammatory treatments have a pronounced effect on survival, which in the pilocarpine model is usually achieved only by stopping SE with diazepam or barbiturates [50]. This may bear significant relevance for the treatment of catastrophic epilepsies in pediatric or adult settings.

It is important to note that a non-inflammatory mechanism for steroidal action exists, namely the modulation of GABA receptors [51]. We are aware of this possible interpretation of results which seems however unlikely given that a number of corticosteroids, ACTH, or IL1-RA exerted the same effects. While our results cannot fully confirm or rule out a specific neuronal action of steroids the fact that the response spanned across a wide range of anti-inflammatory molecules makes this unlikely.

Finally, while steroidal treatment showed promise in our study, the fact remains that the use of potent and potentially harmful anti-inflammatory drugs is not a viable long-term option. The treatment, when successful, had to be repeated once the efficacy waned. This is likely due to traditional, concurrent etiologic mechanisms including cortical dysplasia, other brain lesions, etc. The proposed scenario thus implies that: 1) reduced BBB function on an abnormal cortical/hippocampal background facilitates seizures; 2) The lesional tissue itself promotes BBB dysfunction, cooperating towards a further decrease of the threshold for seizures; 3) Steroids “repair” the BBB while having no impact on circuital and structural abnormalities. This reduces seizure probability; 4) The anti-inflammatory efficacy decreases over time. This vicious cycle, to be interrupted requires simultaneous targeting of neurons and endothelial cells. In multiple drug resistant patients, AEDs are obviously not sufficient to reduce hypersynchronous firing: a new therapy combing anti-inflammatory potency with neuronal targeting may be the necessary and winning combination.

### Conclusions

Our results suggest the potential application of GCs in treating drug resistant seizures and support further studies assessing the effect of GCs in experimental chronic seizures. A prospective randomized trial is needed to elucidate the potential benefit of add-on GCs therapy in drug resistant epilepsy.

## Methods

### Rodents

Rats were housed in a controlled environment (21±1°C; humidity 60%; lights on 08:00 AM - 8:00 PM; food and water available *ad libitum*). Procedures involving animals and their care were conducted in conformity with the institutional guidelines that are in compliance with international laws and policies (EEC Council Directive 86/609, OJ L 358, 1, Dec.12, 1987; Guide for the Care and Use of Laboratory Animals, U.S. National Research Council, 1996). Cleveland Clinic IACUC approved the protocol number 08491 for the performance of the presented experiments.

### Induction of seizures and drug treatments

Rats (male Sprague-Dawley 225–250g) were injected with methylscopolamine (0.5 mg/kg, i.p., Sigma-Aldrich) and 30 minutes after with pilocarpine (340 mg/Kg, Sigma-Aldrich). We have analyzed data obtained from a total of 45 rats (see also Figure S1B). Development of seizure and status epilepticus was evaluated by behavioral (Racine's scale) and EEG assessment. Dexamethasone sodium phosphate (APP Pharmaceutical, IL, USA) was administered 2 mg/kg, i.p. twice a day for 2 days prior scopolamine/pilocarpine treatment. A single dosage of 1 mg/day was also used but did not exert any discernable effects (*data not shown*). IL-RA data included here are relative to [29]. IL-RA was administrated in the tail vein (30 µg/kg) 2 hours before scopolamine/pilocarpine treatment.

### Schedule of treatment-sampling and sacrifice

The number of rats used, timing of drug treatments, blood drawings-analysis and animal sacrifice are indicated in Figure S1. Note that, blood samples were taken at baseline, after each treatment and immediately before (or at onset) of status epilepticus (SE), as evaluated by EEG and behavioral (Racine's Scale) assessments. This methodology has been used in the past by us and other to evaluate the presence of BBB damage and inflammatory process preceding seizure onset [24], . Rats that developed SE despite of dexamethasone pre-treatment were either sacrificed at SE onset to determine BBB damage and serological correlates or followed by EEG/behavioral analysis up to 12 hours (seizures were not stopped with barbiturate) to analyze seizures severity and mortality rate (see Figure S1A).

### Rodent EEG recording and video monitoring

Stereotactic electrode implantation was performed in rats under isofluorane anaesthesia, using the Kopf stereotactic frame. Approximately, half of the rats used were implanted. Four stainless steel screws (MX-0090-2, Small Parts Inc., Miami, Florida) were placed bilaterally on the dura mater of the fronto-parietal cortex. A prefabricated Pinnacle pre-amplifier was connected to the screws. The system has three bio-potential channels - 2 EEG and 1 EMG. Prefabricated head implants (Pinnacle Inc., USA) ensured accurate electrode positioning and reliable, robust contacts. Cable artifacts are eliminated by pre-amplification of the EEG and EMG waveforms at the animal's head. EEG data were sampled a rate of 200 Hz. All data are transferred via a USB connection to a PC. Rats were left unrestrained for 2 weeks to recovery from surgery before EEG recordings were performed. Each rat was kept in a separate cage under 12-hours dark-light cycles with free access to food and water. Origin Microcal 7.0 and Diadem (National Instruments) was used in conjunction to the acquisition system for data analysis (*e.g.,* time-joint frequency analysis).

### FACS and IL-1β ELISA

The schedule of blood sampling is illustrated in Figure S1A. Blood was collected from the tail vein using a fixed catheter (Venisystem, Abbocath, 22G, 300–500 µl/sample). For FACS analysis, 150 µL of whole blood were incubated with a combination of specific antibodies recognizing T-cell subpopulation (CD3+). In particular mouse anti-rat CD8a-FITC, CD3-R-PE and CD4-PE-Cy5 (BD-Pharmingen) were added (2 µL, 5 µL and 5 µL respectively) to blood samples. Blood samples were analyzed by the FACS-Core facility at the Cleveland Clinic. IL-1β ELISA–tests was purchased from Pierce Biotechnology Inc. and performed as described by the vendor.

### Blood-brain barrier integrity assessment

BBB status was assessed using two independent modalities: serum S100β and brain Evan's Blue extravasation [29], [32]. Blood samples were obtained from the tail vein and brains were collected after sacrifice (control, dexamethasone-treated or at onset of SE, n = 5 rats/group). *S100β*: blood samples were collected at the times indicated in Figure S1. Blood samples were centrifuged at 1,200×*g* for 10 min, and the supernatant serum stored at −80°C. The S100β concentration was measured using the Sangtec 100 ELISA method (Diasorin, Stillwater, MN, U.S.A.). *Evan's Blue*: we evaluated the pattern of BBB leakage measuring fluorescent signal present in the brain. The fluorescent solution was prepared reconstituting 2 g of Evans Blue in 100 ml of phosphate buffered saline (0.1 M PBS). The solution was stirred at room temperature in the dark. The solution was infused in the left heart ventricle (2 ml/rat at rate of 1 ml/min). Presence of gross leakage in treated and control animals was evaluated by fluorescent in vivo imaging signaling (IVIS). Brains were placed in the IVIS chamber and background fluorescence was set as zero. Digitalized signals were created following a blue-to-red scale based on the regional distribution of fluorescent signal.

### Human subjects

The study was conducted according to the Declaration of Helsinki Criteria and to the procedures for compassionate drug administration approved by the Ethic Committees of Carlo Besta Institute (Milan, Italy). Oral informed consent from was obtained from parents by specialized neurologists also author of this manuscript. Administration of GCs was considered compassion care since all patients had intractable life threatening seizures. We reviewed the clinical data of 43 patients (Table S1) of the Carlo Besta Neurological Institute (Milan, Italy). All the patients had a known history of intractable seizures and antiepileptic treatment with conventional AEDs was administered (Table S1). We excluded patients with a history of epileptic syndromes known to respond to steroids (*i.e.,* West, Lennox-Gastaut, Landau-Kleffner and Rasmussen encephalitis) as well as patients affected by documented inflammatory brain disease. All the subjects received steroid treatment on an in-patient basis.

Different treatments were employed in different patients: ACTH, dexamethasone, methylprednisolone, and hydrocortisone. Details on glucocorticoids (GCs) or ATCH dosage are summarized in Table S1. Patients received GCs or ACTH therapy because of one of the following: 1) >50% increase in seizure frequency; 2) development of non-convulsive *status epilepticus*; 3) presence of *epilepsia partialis continua* (EPC). Before GCs or ATCH treatment, patients were evaluated by a team of neurologists, physiologists, and underwent EEG and routine laboratory examinations. Antiepileptic treatments were maintained during the steroid course (Table S1).

Steroidal treatment was, when successful, repeated in case of seizure recurrence. For this reason, the number of treatments reported is greater than the number of patients (treatments = 92, see Table S1). GCs or ATCH therapy was considered successful when: 1) seizure frequency decreased by 50%; 2) *status epilepticus* was stopped; 3) or when *epilepsia partialis continua* (EPC) was stopped/reduced enough to allow voluntary movement in the affected body district. Duration of treatment was limited to acute dosing, which was repeated when the initial response was beneficial. The effects of length of treatments and efficacy/toxicity of chronic use are not presented herein, since this study design only addressed a proof of principle use of steroids in pediatric epilepsy.

### Statistical analysis

Spike detection, spike area and instantaneous frequency calculation were performed using pClamp 9.2. Statistics were performed with aid of Origin 7.0 (Microcal) and Jump 7.0; data were considered to be significantly different when p<0.05 (by ANOVA or paired t-test for multiple comparisons). Normal distribution of data was evaluated with Wilk–Shapiro routine. Mosaic plots were graphed with Jump 7.0 and transferred to CorelDraw as metafiles. The Diadem (National Instruments) package was used to construct time-frequency plots. Fisher exact test was used (Jump 7.0) to evaluate the significance of probability of SE and incidence of mortality between groups of animals.

## Supporting Information

Figure S1 **Experimental procedures.** (A) After drug treatment, rats were sacrificed either at SE onset (*e.g.*, to evaluate BBB integrity and FACS -IL-1β analysis) or after 12 hours (*e.g.,* to evaluate EEG changes and mortality). (B) The total number of rats used and the detailed treatment schedule is provided. IL-RA data are relative to [29] **.** See also Methods Section.
(TIF)

Figure S2 **Number of events, peak area and instantaneous frequency distribution.** EEG traces from pilocarpine alone, dexamethasone, or IL-RA pretreated were analyzed using the event detection routine in ClampFit 9.2. Event threshold was set as 2X baseline across all traces. Analysis of typical traces is provided. Different treatments are indicated by different colors. Pilocarpine SE was characterized by the highest frequencies of events. Spike area (time x amplitude) was also greater compared to dexamethasone (red). (B) IL-RA pre-treatment lead to qualitatively and quantitatively similar results.
(TIF)

Figure S3 **Summary of the efficacy of glucocorticosteroids (dexamethasone, methylprednisolone and hydrocortisone) and ACTH in drug resistant pediatric epilepsy.** (A) A total of 92 treatments were evaluated. Treatments were administered as described in the Methods and Table S1. Seizures were assessed by behavioral and EEG observations. The values reported refer to decrease in seizure burden compared to baseline. (B) Mosaic plot showing the correlation between etiology of epilepsy and likelihood of a response ≥50%. C) Although GCs and ACTH were effective across all epileptic syndromes, seizure reduction was more prominent in focal epilepsy patients. D) Therapeutic response (set as ≥50%) did not correlate with seizure history.
(TIF)

Figure S4 **Summary of a multivariate analysis of patients' data, serological measurements and drug efficacy.** Significant p value (<0.05) is indicated by a red square. Among the variables analyzed the following are here described: 1) age was not a factor influencing GCs or ACTH efficacy; 2) a trend toward significance was observed for the following pairs: efficacy and number of neutrophils, efficacy and number of WBC. A larger population study is required to assess full significance of leukocytes variation in relation to seizure burden and reduction.
(TIF)

Table S1 Summary of Patients' data.
(DOC)
