# Supplementary figures and images for: Efficacy of Anti-Inflammatory Therapy in a Model of Acute Seizures and in a Population of Pediatric Drug Resistant Epileptics
Source: PLoS One. 2011 Mar 28;6(3):e18200. doi: 10.1371/journal.pone.0018200 (PMC3065475; doi:10.1371/journal.pone.0018200)

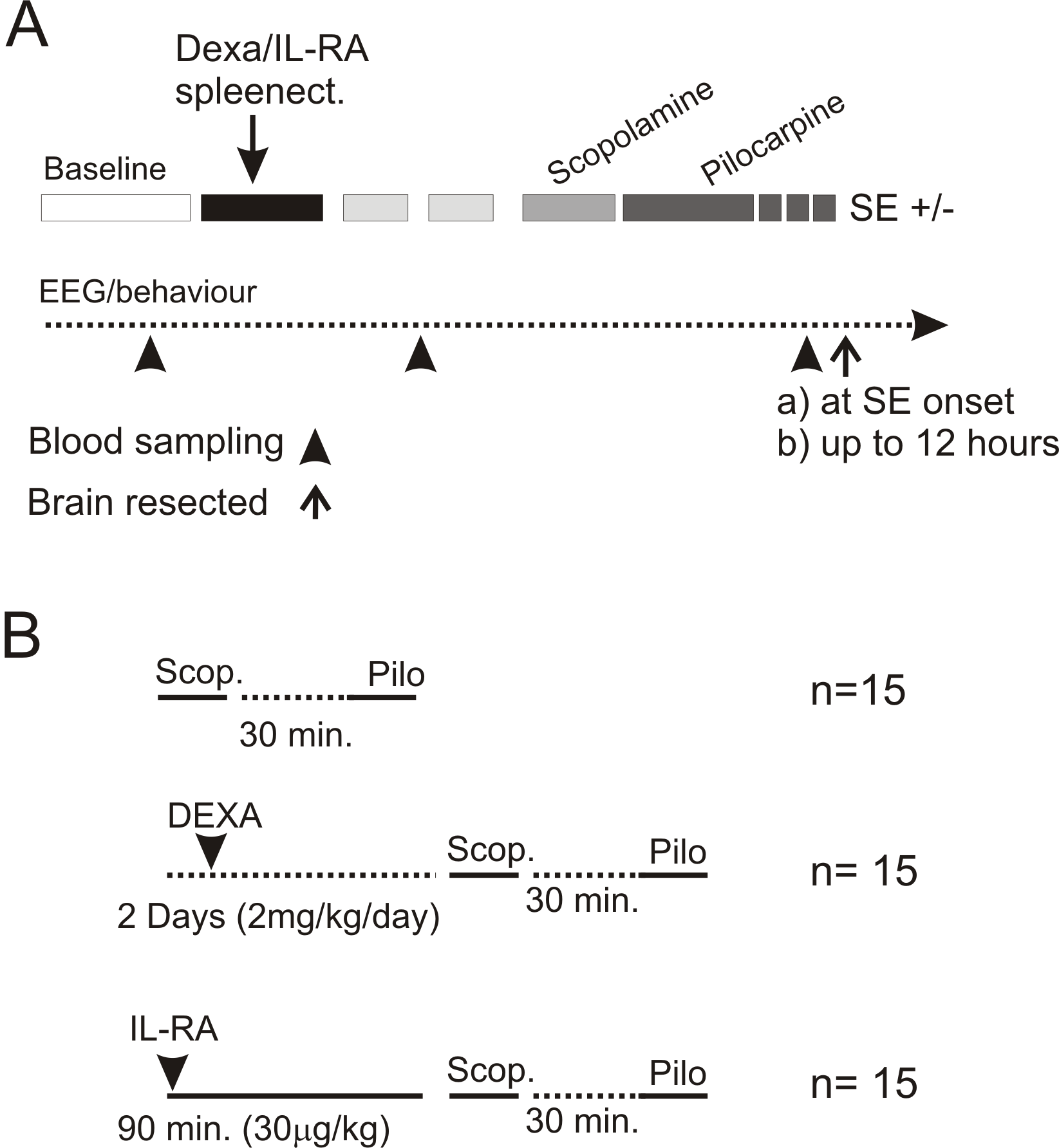

Supplement: Figure S1 — Experimental procedures. (A) After drug treatment, rats were sacrificed either at SE onset (e.g., to evaluate BBB integrity and FACS -IL-1β analysis) or after 12 hours (e.g., to evaluate EEG changes and mortality). (B) The total number of rats used and the detailed treatment schedule is provided. IL-RA data are relative to [29] . See also Methods Section. (TIF) [file pone.0018200.s001.tif]

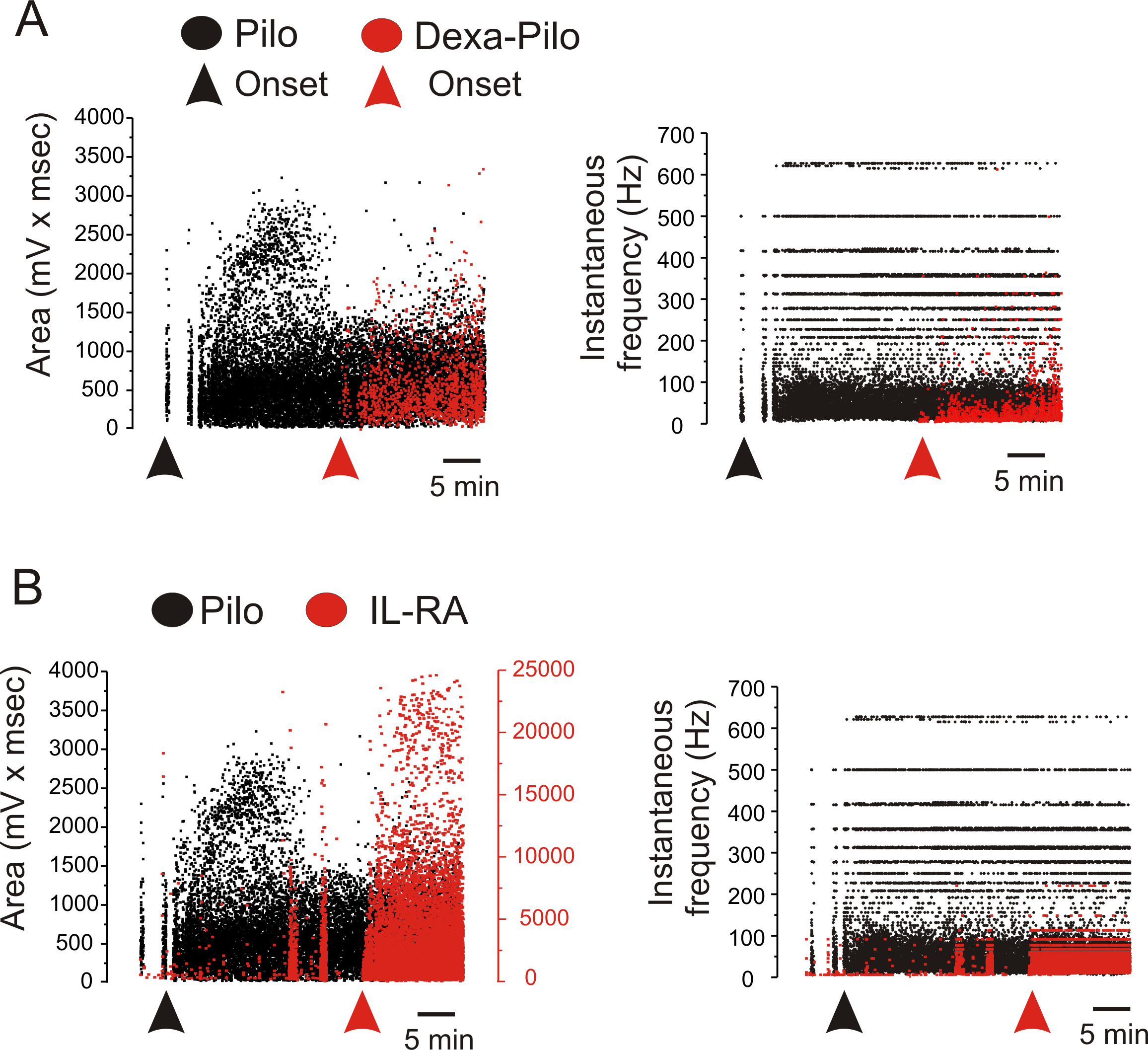

Supplement: Figure S2 — Number of events, peak area and instantaneous frequency distribution. EEG traces from pilocarpine alone, dexamethasone, or IL-RA pretreated were analyzed using the event detection routine in ClampFit 9.2. Event threshold was set as 2X baseline across all traces. Analysis of typical traces is provided. Different treatments are indicated by different colors. Pilocarpine SE was characterized by the highest frequencies of events. Spike area (time x amplitude) was also greater compared to dexamethasone (red). (B) IL-RA pre-treatment lead to qualitatively and quantitatively similar results. (TIF) [file pone.0018200.s002.tif]

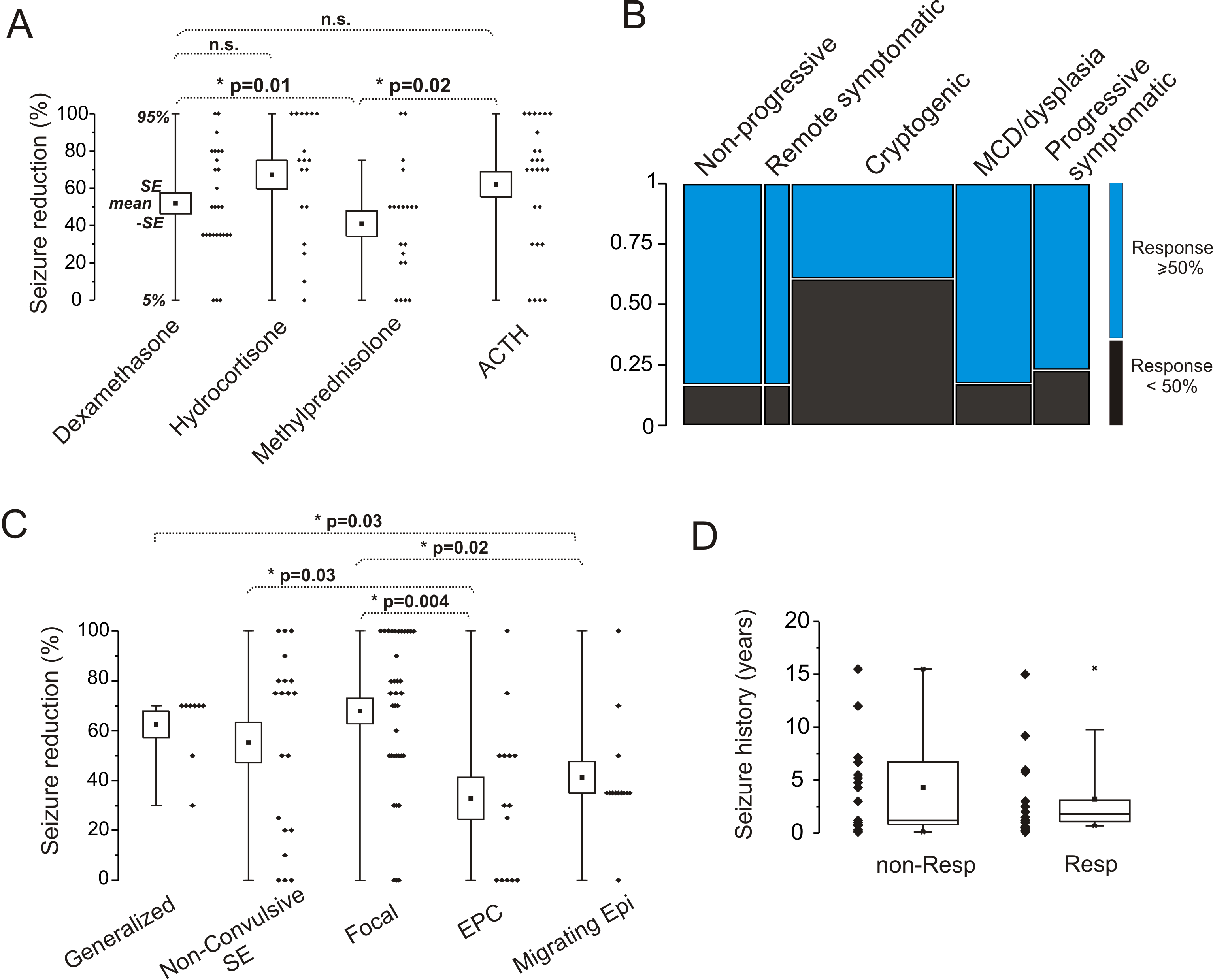

Supplement: Figure S3 — Summary of the efficacy of glucocorticosteroids (dexamethasone, methylprednisolone and hydrocortisone) and ACTH in drug resistant pediatric epilepsy. (A) A total of 92 treatments were evaluated. Treatments were administered as described in the Methods and Table S1. Seizures were assessed by behavioral and EEG observations. The values reported refer to decrease in seizure burden compared to baseline. (B) Mosaic plot showing the correlation between etiology of epilepsy and likelihood of a response ≥50%. C) Although GCs and ACTH were effective across all epileptic syndromes, seizure reduction was more prominent in focal epilepsy patients. D) Therapeutic response (set as ≥50%) did not correlate with seizure history. (TIF) [file pone.0018200.s003.tif]

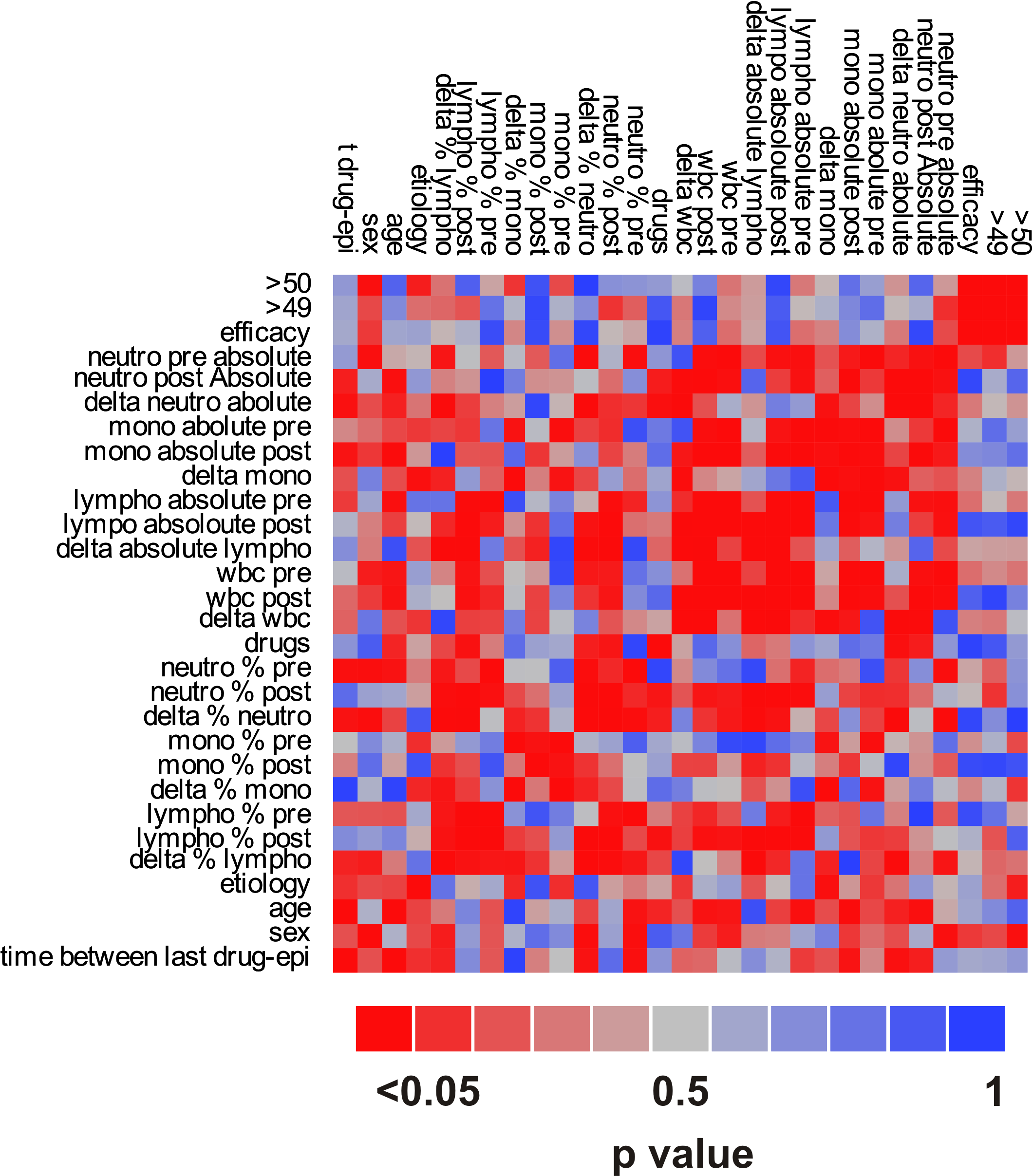

Supplement: Figure S4 — Summary of a multivariate analysis of patients' data, serological measurements and drug efficacy. Significant p value (<0.05) is indicated by a red square. Among the variables analyzed the following are here described: 1) age was not a factor influencing GCs or ACTH efficacy; 2) a trend toward significance was observed for the following pairs: efficacy and number of neutrophils, efficacy and number of WBC. A larger population study is required to assess full significance of leukocytes variation in relation to seizure burden and reduction. (TIF) [file pone.0018200.s004.tif]
