# Supplementary material for: Efficacy of Anti-Inflammatory Therapy in a Model of Acute Seizures and in a Population of Pediatric Drug Resistant Epileptics
Source: PLoS One. 2011 Mar 28;6(3):e18200. doi: 10.1371/journal.pone.0018200 (PMC3065475; doi:10.1371/journal.pone.0018200)
Supplement: Table S1 — Summary of Patients' data. (DOC) [file pone.0018200.s005.doc]

| **ID** | **Sex** | **Seizure Type** | **Type of Epilepsy** | **Etiology** | **AED** | **CGs/**  **ACTH** | **Dose**  **kg/day** | **Age at treat. (y,m)** | **Efficacy**  **%** |
| --- | --- | --- | --- | --- | --- | --- | --- | --- | --- |
| **C.A.1** | F | Partial | EPC | mitocondrial disease | **PB**, VPA, TPM,PHT, **MDZ,** | 2 | 1 mg | 3 | 50 |
| **C.A.2** | F | Partial | EPC | mitocondrial disease | **PB**, VPA, TPM, **LVT**,PHT, **MDZ,** | 3 | 20 mg | 3 | 30 |
| **C.A.3** | F | Partial | EPC | mitocondrial disease | **PB**, VPA, TPM, **LVT**,PHT, **MDZ, CZP** | 3 | 20 mg | 3 | 50 |
| **C.L.** | F | Partial | EPC | genetic non progressive | **CBZ**, TPM, PB, VPA, PHT, GVG, LTG, Clo, CZP, LVT, MDZ, | 3 | 15 mg | 7 | 50 |
| **C.M.** | F | Partial + SG | EPC | unknown | **CBZ**, PB, **VPA**, Clo, **CZP**, **LVT,** MDZ, **ACZ** | 3 | 15 mg | 5.9 | 50 |
| **C.M.1** | F | Partial | EPC | focal dysplasia | **PB**, **PHT**, LTG, **Clo**, **CBZ**, **VPA**, ESM, GVG, **ACZ** | 4 | 3 mg | 4.9 | 100 |
| **C.M.2** | F | Partial | EPC | focal dysplasia | **PB**, **PHT**, LTG, **Clo**, **CBZ**, **VPA**, ESM, GVG, **ACZ** | 2 | 0.44 mg | 4.9 | 75 |
| **D.C.1** | **F** | Partial + SG | EPC | unknown | CBZ,**TPM**,PB,Clo,BR, LVT,ZNS | 3 | 15 mg | 6.1 | 75 |
| **D.C.2** | **F** | Partial + SG | EPC | unknown | CBZ,**TPM**,PB,Clo,BR, LVT,ZNS | 3 | 15 mg | 7 | 0 |
| **D.C.3** | **F** | Partial + SG | EPC | unknown | CBZ,**TPM,PB**,Clo,BR, LVT,ZNS | 3 | 15 mg | 8.8 | 50 |
| **D.C.A** | M | tonic-clonic/  myoclonic/focal | EPC | unknown | **TPM**, Pir, **CZP**, CBZ,PB,Clo, | 3 | 15 mg | 14.1 | 30 |
| **M.T.V.1** | F | Partial | EPC | congenital muscular dystrophy | **PB**,TPM,  **GVG**,Clo,  MDZ,PHT,  BR | 1 | 3 U | 0.6 | 0 |
| **M.T.V.2** | F | Partial | EPC | congenital muscular dystrophy | **PB**,TPM,  **GVG**,**Clo**,  MDZ,PHT,  BR | 2 | 0.2 mg | 0.7 | 0 |
| **P.E.1** | M | Partial/SG | EPC | unknown /progressive | PB, **VPA**, PHT, CBZ, GVG,LTG,  Clo, **CZP**, **LVT**, MDZ, Lor | 2 | 0.45 mg | 7.1 | 0 |
| **P.E.2** | M | Partial/SG | EPC | unknown /progressive | PB, VPA, **PHT**, CBZ, GVG,LTG,  Clo, CZP, LVT, MDZ, Lor,**TGB** | 2 | 0.2 mg | 7.8 | 0 |
| **P.F.** | F | Partial/SG | EPC | unknown | **TPM,** VPA,PHT,  Clo, LVT,**CBZ** | 3 | 15 mg | 8 | 50 |
| **V.J.1** | F | Myoclonic, partial | EPC | unknown /progressive | PB, VPA, PHT, **CBZ**, GVG,**LTG,**  **Clo**, CZP, LVT, MDZ, PRM,TPM, | 3 | 15 mg | 16 | 25 |
| **V.J.2** | F | Myoclonic, partial | EPC | unknown /progressive | **PB,** VPA, PHT, CBZ, GVG,LTG,  Clo, CZP, LVT, MDZ, PRM,**TPM,** | 3 | 15 mg | 16.2 | 0 |
| **B.C.1** | F | Partial/SG | Focal | chromosomopathy | **VPA**, **CZP**,Clo,ESM,LTG,PB,  GVG,CBZ,  TPM | 2 | 0.5 mg | 0.8 | 100 |
| **B.C.2** | F | Partial/SG | Focal | chromosomopathy | **VPA,** CZP,Clo,ESM,LTG,PB,  GVG,CBZ,  TPM | 4 | 7.5 mg | 0.9 | 100 |
| **B.L.1** | M | Partial/  myoclonic | Focal | cerebral palsy | PB,**VPA**,Clo,CBZ,ESM,  LVT,LTG | 4 | 5 mg | 6.3 | 75 |
| **B.L.2** | M | Partial/  myoclonic | Focal | cerebral palsy | PB,**VPA**,**Clo**,CBZ,**ESM**,  LVT,LTG | 4 | 5 mg | 7.8 | 100 |
| **B.L.3** | M | Partial/  myoclonic | Focal | cerebral palsy | PB,**VPA,Clo**,CBZ,**ESM**,  LVT,LTG | 1 | 2.6 U | 8 | 50 |
| **B.V.1** | F | Partial/SG/spasms | Focal | unknown | PB, **VPA**, CBZ, GVG,LTG,  Clo, CZP, LVT, PRM,TPM, ESM, BR, KD | 1 | 3.6 U | 1.3 | 100 |
| **B.V.2** | F | Partial/SG/spasms | Focal | unknown | PB, **VPA,** CBZ, **GVG**,LTG,  Clo, CZP, LVT, PRM,TPM, ESM, BR, KD | 3 | 2 mg | 3 | 0 |
| **C.A.** | M | Partial/SG | Focal | unknown | VPA,PB,**CBZ,TPM**, LTG | 1 | 4.7U | 3.8 | 100 |
| **C.F.** | F | Partial/  myoclonic | Focal | genetic non progressive | B6, **GVG,VPA,**  LTG,Clo | 1 | 3 U | 1.5 | 75 |
| **C.L.1** | M | Partial/  myoclonic | Focal | genetic non progressive | CZP, **VPA**,Clo,  GVG,ESM,  CBZ,LTG, MSM | 4 | 10 mg | 3 | 100 |
| **C.L.2** | M | Partial/  myoclonic | Focal | genetic non progressive | CZP, **VPA**,Clo,  GVG,ESM,  CBZ,LTG, MSM | 4 | 10 mg | 3.5 | 100 |
| **C.L.3** | M | Partial/  myoclonic | Focal | genetic non progressive | CZP, **VPA,**Clo,  GVG,ESM,CBZ,**LTG**, MSM | 4 | 5 mg | 7.2 | 100 |
| **C.M.1** | F | Partial/SG | Focal | focal dysplasia | **VPA**,ESM,LVT,Clo,TPM,BR,CBZ,PHT,PB | 2 | 0.1 mg | 6.9 | 90 |
| **C.M.2** | F | Partial | Focal | focal dysplasia | **VPA**,ESM,LVT,Clo,TPM,BR,CBZ,PHTPB | 2 | 0.1 mg | 6.9 | 70 |
| **C.M.3** | F | Partial | Focal | focal dysplasia | **VPA**,ESM,**LVT,Clo**,TPM,BR,CBZ,  PHT,PB | 4 | 8 mg | 8.4 | 50 |
| **C.M.4** | F | Partial/SG | Focal | focal dysplasia | **VPA,ESM**,LVT,Clo,TPM,BR,CBZ,PHTPB | 4 | 8.3 mg | 8.4 | 0 |
| **C.R.C.1** | F | Partial + SG | Focal | focal dysplasia | **VPA**,GVG,CBZ,LTG,**Clo**,**TPM,** | 2 | 0.4 mg | 5.8 | 50 |
| **C.R.C.2** | F | Partial + SG | Focal | focal dysplasia | **VPA**,GVG,CBZ,LTG,**Clo**,**TPM,** | 2 | 0.4 mg | 6 | 50 |
| **C.R.C.3** | F | Partial + SG | Focal | focal dysplasia | VPA,GVG**,**  **CBZ**,LTG,  Clo,TPM, LVT, ZNS, RFN,**PB** | 2 | 1 mg | 8.6 | 70 |
| **D.M.A** | F | Partial with sporadic status | Focal | unknown | CBZ,LVT,  Clo,PB,MDZ,**Lor, SUL**, **PHT,** | 3 | 15 mg | 13 | 70 |
| **F.A.** | M | Partial/  tonic | Focal | Krabbe disease, stem cell transplantation | **VPA,ESM**,  LTG | 2 | 0.2 mg | 7.2 | 80 |
| **I.N.** | F | Myoclonic, partial | Focal | Krabbe disease, stem cell transplantation | **VPA,CBZ**,  PRM,LVT,  PB | 3 | 10 mg | 8.8 | 100 |
| **M.T.** | M | Partial | Focal | focal dysplasia |  | 3 | 30 mg | 3.9 | 0 |
| **R.S.1** | F | Partial | Focal | focal dysplasia | **GVG, PHT**, PB, PRM, Clo, CBZ, VPA | 2 | 0.3 mg | 0.5 | 80 |
| **R.S.2** | F | Partial | Focal | focal dysplasia | GVG, PHT, PB, **PRM, Clo**, CBZ, VPA | 4 | 10 mg | 1 | 80 |
| **V.A.** | M | Partial/SG | Focal | diffuse cortical dysplasia | PB,**VPA,**  **PHT**,GVG,  LTG,BR,**Clo**,LVT,TPM,  KD | 1 | 2.6 U | 14 | 100 |
| **V.E.** | F | Partial + SG | Focal | unknown | CBZ,VPA,  Clo,LTG,  GVG,PHT,  PRM,PB,  FBM,LVT,  TPM, | 2 | 0.2 mg | 17 | 80 |
| **V.M.1** | M | Partial/  tonic/ absences | Focal | unknown | PB,TPM,  CBZ,LTG,  GVG,PRM, FBM,**VPA**,  ESM,PHT,  CBZ,Clo, ZNS | 4 | 10 mg | 1.3 | 30 |
| **V.M.2** | M | Partial/  tonic/ absences | Focal | unknown | PB,TPM,  CBZ,LTG,  GVG,PRM, FBM,VPA,  ESM**,PHT**,  CBZ,**Clo,** ZNS | 1 | 4 U | 2.7 | 30 |
| **V.M.3** | M | Partial/  tonic/ absences | Focal | unknown | PB,TPM,  **CBZ,LTG**,  GVG,PRM, FBM,**VPA**,  ESM,PHT,  CBZ,Clo, ZNS | 1 | 2 U | 13.8 | 30 |
| **M.M.** | M | Partial/  myoclonic | Focal | cerebral palsy | CBZ,GVG,  **VPA,Clo,**  ESM,**LTG**,PRM, **PHT,** TPM | 2 | 0.1 mg | 18.3 | 60 |
| **P.E.** | F | Partial  multifocal | Focal | unknown /non progressive | **CBZ,TPM,**  **PB**,LTG,Clo,GVG, **MDZ** | 3 | 15 mg | 14 | 100 |
| **C.G.1** | M | Myoclonic, absence, SG | Generalized | mitocondrial disease | CBZ, VPA, Clo, **FBM**, ESM, LVT, PRM, **NTZ** | 1 | 0.7 U | 14.7 | 70 |
| **C.G.2** | M | Myoclonic, absence, SG | Generalized | mitocondrial disease | CBZ, VPA, Clo, **FBM**, ESM, LVT, PRM, **NTZ** | 1 | 1.5 U | 14.8 | 70 |
| **C.G.3** | M | Myoclonic, absence, SG | Generalized | mitocondrial disease | CBZ, VPA, Clo, **FBM**, ESM, LVT, PRM, **NTZ** | 4 | 4.2 mg | 15.3 | 70 |
| **C.G.4** | M | Myoclonic, absence, SG | Generalized | mitocondrial disease | CBZ, VPA, Clo, **FBM**, ESM, LVT, PRM, **NTZ** | 1 | 1.5 U | 15.6 | 70 |
| **C.G.5** | M | Myoclonic, absence, SG | Generalized | mitocondrial disease | CBZ, VPA, Clo, **FBM**, ESM, LVT, PRM, **NTZ** | 1 | 1.35 U | 18.4 | 70 |
| **C.G.6** | M | Myoclonic, absence, SG | Generalized | mitocondrial disease | CBZ, **VPA**, Clo, FBM, **ESM**, LVT, PRM, | 1 | 1.35 U | 22 | 70 |
| **I.P.** | M | Generalized  myoclonic | Generalized | unknown | **VPA,** Clo, **ESM**, LTG, LVT | 1 | 2.9 U | 3.1 | 30 |
| **V.A.** | F | myoclonic | Generalized | genetic non progressive | **VPA,ESM** | 1 | 3 U | 2.7 | 50 |
| **S.A.1** | F | Partial (multifocal) | MMPEI | unknown | PB, GVG, **PHT**, VPA, CBZ, CNZ | 2 | 0.65 mg | 0.6 | 35 |
| **S.A.2** | F | Partial (multifocal) | MMPEI | unknown | PB, GVG, **PHT**, VPA, CBZ, CNZ | 2 | 0.65 mg | 0.6 | 35 |
| **S.A.3** | F | Partial (multifocal) | MMPEI | unknown | PB, GVG, PHT, **VPA,** CBZ, CNZ | 2 | 0.75 mg | 0.6 | 35 |
| **S.A.4** | F | Partial (multifocal) | MMPEI | unknown | PB, GVG, PHT, **VPA,** CBZ, CNZ | 2 | 0.75 mg | 0.6 | 35 |
| **S.A.5** | F | Partial (multifocal) | MMPEI | unknown | PB, GVG, PHT, **VPA,** CBZ, CNZ | 2 | 0.75 mg | 0.6 | 35 |
| **S.A.6** | F | Partial (multifocal) | MMPEI | unknown | PB, GVG, PHT, **VPA,** CBZ, CNZ | 2 | 0.75 mg | 0.9 | 35 |
| **S.A.7** | F | Partial (multifocal) | MMPEI | unknown | PB, GVG, PHT, **VPA,** CBZ, CNZ | 2 | 0.75 mg | 0.9 | 35 |
| **S.A.8** | F | Partial (multifocal) | MMPEI | unknown | PB, GVG, PHT, **VPA,** CBZ, CNZ | 2 | 0.75 mg | 0.9 | 100 |
| **S.A.9** | F | Partial (multifocal) | MMPEI | unknown | PB, GVG, PHT, **VPA,** CBZ, CNZ | 2 | 0.75 mg | 0.9 | 35 |
| **S.A.10** | F | Partial (multifocal) | MMPEI | unknown | PB, GVG, PHT, **VPA,** CBZ, CNZ | 2 | 0.75 mg | 0.9 | 35 |
| **Z.R.1** | M | Partial (multifocal) | MMPEI | unknown | PB,ACTH,  GVG,**LTG**,  CBZ,**VPA**,CNZ, BR | 4 | 10 mg | 2.1 | 70 |
| **Z.R.2** | M | Partial (multifocal) | MMPEI | unknown | PB,ACTH,  GVG,**LTG**,  CBZ,**VPA**,  CNZ, BR | 2 | 0.2 mg | 2.8 | 50 |
| **A.F.** | M | Myoclonic, partial | non conv. ES | genetic non progressive | **VPA**,Clo,  LVT,**LTG** | 1 | 3.5 U | 4.1 | 100 |
| **A.S.** | F | Partial (multifocal) | non conv. ES | diffuse cortical dysplasia | **VPA,ESM,** Clo,LTG | 1 | 3 U | 6 | 90 |
| **C.C.1** | F | Tonic/  spasms/focal | non conv. ES | unknown | **PB**, VPA, Clo, GVG, TPM, LTG, DZP, CNZ, BR, LVT | 1 | 5 U | 0.4 | 80 |
| **C.C.2** | F | Tonic/  spasms/focal | non conv. ES | unknown | PB, VPA, **Clo**, GVG, **TPM**, LTG, DZP, CNZ, BR, LVT | 4 | 5 mg | 4.5 | 25 |
| **C.C.3** | F | Tonic/  spasms/focal | non conv. ES | unknown | **PB,** VPA, Clo, GVG, TPM, LTG, DZP, CNZ, BR, LVT | 1 | 2.6 U | 6.9 | 75 |
| **C.F.1** | M | Partial | non conv. ES | unknown | CBZ,**VPA**,  TPM,LVT,  Clo,MDZ, ESM, DZP, **LTG** | 3 | 15 mg | 4.6 | 20 |
| **C.F.2** | M | Partial | non conv. ES | unknown | CBZ,**VPA,**  **TPM**,LVT,  Clo, MDZ, ESM, DZP | 1 | 2.5 U | 4.6 | 0 |
| **G.M.1** | F | Partial/  myoclonic | non conv. ES | Post-surgical | PB, **CZP,** CBZ, PRM, **VPA**, ESM, LTG | 3 | 10 mg | 16 | 50 |
| **G.M.2** | F | Partial/  myoclonic | non conv. ES | Post-surgical | PB, **CZP,** CBZ, PRM, **VPA, ESM,** LTG | 3 | 15 mg | 16 | 20 |
| **M.M.** | M | tonic | non conv. ES | genetic non progressive | **VPA,**ESM,  LTG,CZP,  LVT,SUL,  **TPM,** | 1 | 2.5 U | 6 | 100 |
| **M.P.1** | M | Partial/  myoclonic | non conv. ES | genetic non progressive | **VPA, ESM**, Clo, LVT, TPM, LTG, CNZ, BR, PB | 1 | 3 U | 4 | 80 |
| **M.P.2** | M | Myoclonic | non conv. ES | genetic non progressive | **VPA, ESM**, Clo, LVT, TPM, LTG, CNZ, BR, PB | 1 | 3 U | 4.3 | 75 |
| **M.P.3** | M | Myoclonic | non conv. ES | genetic non progressive | **VPA, ESM**, Clo, LVT, TPM, LTG, CNZ, BR, PB | 1 | 3 U | 4.8 | 0 |
| **M.P.4** | M | Partial/  myoclonic | non conv. ES | genetic non progressive | VPA, ESM, Clo, LVT, TPM, LTG, CNZ, BR, **PB** | 1 | 3 U | 5 | 0 |
| **N.M.1** | M | Partial | non conv. ES | diffuse cortical dysplasia | **VPA,** CBZ, **LTG**, PHT, TPM, PB, ESM | 4 | 10 mg | 2 | 75 |
| **N.M.2** | M | Partial | non conv. ES | diffuse cortical dysplasia | **VPA,** CBZ, **LTG**, PHT, TPM, PB, ESM | 4 | 10 mg | 2.4 | 50 |
| **S.K.1** | M | myoclonic | non conv. ES | genetic non progressive | CPA, CNZ, PB,LVT,  **ESM**,Clo,  LTG, **VPA** | 1 | 2.6 U | 5.9 | 100 |
| **S.K.2** | M | myoclonic | non conv. ES | genetic non progressive | CPA, CNZ, PB,LVT,  ESM,**Clo**,  LTG, **VPA** | 4 | 5 mg | 6.5 | 10 |
| **S.K.3** | M | myoclonic | non conv. ES | genetic non progressive | CPA, CNZ, PB,LVT,  **ESM**,**Clo**,  **LTG, VPA** | 4 | 2.5 mg | 6.9 | 75 |
| **V.C.** | F | Partial/SG | non conv. ES | genetic non progressive | **VPA, Clo**, TPM, LVT, CBZ, GVG | 2 | 0.15 | 7 | 80 |

**Table 1. Summary of Patients’ data**

**1= ACTH; 2= dexamethasone; 3= metilprednisolone; 4= hydrocortisone**

MMPE: malignant migrating partial epilepsy of infancy; SG= secondarily generalized

VPA= valproic acid; PRM= primidone; ESM= Etosuximide; LTG= Lamotrigine; PB= Phenobarbital; CBZ= Carbamazepine; PHT= Phenitoin; MDZ= Midazolam; Clo= Clobazam; CNZ=Clonazepam; NTZ= nitrazepam; SUL= Sulthiame; GVG = vigabatrin; FBM= felbamate; ZNS= zonisamide**. AEDs co-administered with GCs or ACTH are indicated in bold.**

KD=Ketogenic diet; BR= Bromides
